# The Role of AIF-1 in the Aldosterone-Induced Vascular Calcification Related to Chronic Kidney Disease: Evidence From Mice Model and Cell Co-Culture Model

**DOI:** 10.3389/fendo.2022.917356

**Published:** 2022-07-20

**Authors:** Xueying Chang, Jianbing Hao, Xingzhi Wang, Jingwei Liu, Jie Ni, Lirong Hao

**Affiliations:** ^1^ Department of Nephropathy and Hemodialysis, The First Affiliated Hospital of Harbin Medical University, Harbin, China; ^2^ Department of Nephropathy, Southern University of Science and Technology Hospital, Shenzhen, China

**Keywords:** aldosterone, allograft inflammatory factor-1, vascular calcification, chronic kidney disease, cell co-culture

## Abstract

Increasing evidence suggests that aldosterone (Aldo) plays an essential role in vascular calcification which is a serious threat to cardiovascular disease (CVD) developed from chronic kidney disease (CKD). However, the exact pathogenesis of vascular calcification is still unclear. First, we established CKD-associated vascular calcification mice model and knockout mice model to investigate the causal relationship between allograft inflammatory factor 1 (AIF-1) and vascular calcification. Then, endothelial cells (ECs) and vascular smooth muscle cells (VSMCs) co-culture experiments were performed to further explore the mechanisms of calcification. The results of the Aldo intervention mice model and transgenic mice model showed that Aldo could cause calcification by increasing the AIF-1 level. The results of *in vitro* co-culture model of ECs and VSMCs showed that AIF-1 silence in ECs may alleviate Aldo-induced calcification of VSMCs. In conclusion, our study indicated that Aldo may induce vascular calcification related to chronic renal failure *via* the AIF-1 pathway which may provide a potential therapeutic target.

## Introduction

Chronic kidney(CKD) disease with vascular calcification is a strong predictor of cardiovascular disease (1, 2). Understanding the possible etiology and mechanism of vascular calcification is of great significance for reducing the incidence and mortality of cardiovascular and cerebrovascular events in patients with CKD (3, 4) As known, aldosterone (Aldo) is known to be a participant in the metabolites of the renin-angiotensin- Aldo system, which not only regulates blood volume and consequently blood pressure homeostasis by regulating sodium retention, but also stimulates the process of vascular calcification (5, 6). Evidence has shown the plasma Aldo level in patients with CKD is significantly increased, and with the increase of plasma Aldo level, the occurrence of vascular calcification is on the rise (7). Somatic gene mutations that favor the inappropriate entry of intracellular calcium have been found in aldosterone-producing adenomas (APA) (8). The expression of local Aldo synthase and Aldo receptor was significantly enhanced in uremic vascular calcification rats and was positively correlated with the degree of vascular calcification, suggesting that local Aldo spontaneous synthesis plays an important role in vascular calcification in CKD (9).

Vascular calcification is an active and regulated process involving many types of cells, such as endothelial cells (ECs), vascular smooth muscle cells (VSMCs), and inflammatory cells, and all the three cells interact through complex signaling pathways in the vascular micro-environment (10, 11). Once inflammation occurs, the stability of ECs will be destroyed, and numerous inflammatory mediators are produced and immersed in the vascular tissue, damaging the VSMCs in the middle membrane and leading to vascular calcification (12). In addition, VSMCs can promote vascular calcification through osteochondral transdifferentiation, extracellular vesicle release, and apoptosis (13). Considerable evidence suggests that the interaction between ECs and VSMCs may promote the occurrence of vascular calcification under undesirable stimulation, but the mechanism of ECs injury-induced calcification of the membrane VSMCs is not yet clear. A better understanding of the relationship between ECs, VSMCs, and vascular calcification could be important for the prevention and treatment of vascular calcification.

Allograft inflammatory factor-1 (AIF-1) is an important cytokine in the process of the inflammatory response, which could affect the production of cytokines and the expression of inflammatory mediators (14, 15). It has been found that cytosolic factors P42/44 and PAK1 could significantly stimulate AIF-1 transcription, thus affecting translation, after ECs inflammatory stimulation, which could promote the proliferation and migration of ECs, and play a role in activating cascade-amplifying inflammatory response and vascular injury (16). And the expression of AIF-1 could also increase the osteogenic transdifferentiation in VSMCs (17). Moreover, previous studies showed Aldo could induce a severe coronary artery perivascular inflammatory response through inflammatory mediators and perivascular fibrosis (18, 19). However, very few studies have reported the effects of Aldo and AIF-1-mediated interactions between ECs and VSMCs on vascular calcification. Therefore, we hypothesized that Aldo in the CKD microenvironment might lead to VSMCs calcification by stimulating the increased AIF-1 expression in ECs, resulting in secondary downstream inflammation. To verify the hypothesis, this study used a CKD and AIF-1 knockout(AIF-1KO) mice model to clarify the relationship between AIF-1 and vascular calcification in Aldo-induced CKD. And through the co-culture of cells, the role and mechanism of AIF-1 in ECs leading to calcification of VSMCs were further explored, aiming to provide a new theoretical basis and potential therapeutic targets for vascular calcification in CKD.

## Method

### CKD-Associated Vascular Calcification Mice Model and AIF-1 Knockout Mice Model

In the chronic kidney disease mouse model, 24 male C57BL/6J mice (aged 8-10 weeks) were randomly divided into four groups: control group, CKD group, CKD+Spironolactone (Spir) group, and Spir group, with six mice in each group(purchased from Beijing Vital River Laboratory Animal Technology Co. Ltd); in the AIF-1 knockout(AIF-1KO) mouse model, 12 male C57BL/6J mice and 12 male AIF-1KO mice (purchased from the Center for Biochemistry and Molecular Biology in Harbin Medical University; generated through CRISPR/Cas9 system: aged 8-10 weeks) were randomly divided into four groups: control group, CKD group, AIF-1KO group, and AIF-1KO+CKD group. Spir was purchased from Sigma Aldrich, and additional reagents and resources are listed in **Supplementary Table 1**. The mice in the CKD group and CKD+Spir group were given special feed containing 0.25% adenine, 0.8% phosphorus, and 5% casein in the first four weeks and given special feed containing 0.25% adenine, 1.8% phosphorus, 5% casein in the last four weeks to induce vascular calcification in chronic kidney disease, and other groups were fed 5% casein diet. And to investigate the role of the AIF-1 gene in vascular calcification, 12 male C57BL/6J mice and 12 male AIF-1KO mice were randomly divided into four groups: control group, Aldo CKD group, AIF-1KO+CKD group, AIF-1KO group, with six mice each group. The mice in the control and AIF-1KO groups were fed with the standard food containing 5% casein, and the mice in the CKD group, and AIF-1KO+CKD group were treated with the special feed as the method described above. All the mice were sacrificed with CO2 euthanasia after treatment at the age of 16-18 weeks, and the serum and aortic vessels were collected. All mice were fed in the SPF animal center at a temperature of 15°C-25°C, a humidity of (75 ± 15) %, and a light/darkness cycle for 12 h, feeding freely with a standard pellet. The animal experiments were performed according to the protocols approved by the Animal Ethics Review Committee of Harbin Medical University and adhered to the principles stated in the Guide for the Care and Use of Laboratory Animals.

### Histopathological Evaluation

The thoracic aorta and kidneys of mice were isolated and placed into a 4% paraformaldehyde fixative solution for 24 h. Then it was dehydrated in a graded series of ethanol (50%, 12 h; 70%, 2 h; 85%, 2 h; 95%, 2 h; 100%, 2 h; 100%, 2 h), cleared in a mixture solution of xylene and anhydrous ethanol (1:1), and embedded in paraffin for 1 h. Serial slices were cut using a microtome at a thickness of 8 μm. After staining with hematoxylin and eosin, the slices were observed and photographed slices with an optical microscope (Olympus, Tokyo, Japan).

### Vascular Calcification Staining

VonKossa staining was performed to detect calcium deposition in mouse arteries. Arterial sections were dewaxed, washed in water, and then irradiated with 1% silver nitrate solution under UV light for 45 min, followed by staining of the nucleus and cytoplasm with hematoxylin and eosin, respectively. The areas of calcium deposits in the vessels showed black staining when observed under light microscopy.

### Measurement of Serum Cr, BUN, Ca^2+^, AIF-1, Aldo, PTH, FGF23, and Phosphorus in Mice

Blood samples in mice were collected and centrifuged at 3000 rpm for 15 min. Serum Cr, BUN, Ca^2+^, and phosphorus were detected by an automatic biochemical analyzer. Serum AIF-1, PTH, FGF23, and Aldo were measured using corresponding commercial kits according to the manufacturer’s protocol. The absorbance was measured at 450 nm and the average value was calculated to analyze the concentration of the indicator through the standard curve.

### Co-Culture of ECs With VSMCs

To better simulate the physiological environment of the vessel wall, ECs and VSMCs co-culture experiments were performed in line with the previous studies (20, 21). The ECs and VSMCs were respectively purchased from American Type Culture Collection and Beina Chuanglian Biotechnology Co., Ltd. After AIF-1 transfection, ECs were added with 10^-8^ mmol/L Aldo and cultured at 37°C or 48 h and collected the supernatant. VSMCs were laid in 24-well plates with cell slivers and cultured with a medium containing 1.5 mmol/L calcium chloride for 48 hours. Then the supernatant of ECs was added to co-culture for 5 days in the NC group, shAIF- 1 group, Aldo+shAIF- 1 group, and Aldo+NC group.

### Flow Cytometric Measurement of Calcium Ion Concentration in VSMCs

The VSMCs were cultivated on a 96-well plate. After incubation for 24 h, the cell medium was removed. The cell suspension was added with Fluo-3/Am solution to 10 umol/L and incubated in a 37°C carbon dioxide incubator for 1 h, with slight shaking 3 times during the incubation. The cells were centrifuged and washed with PBS. The fluorescence intensity of FluO-3 binding to intracellular Ca^2+^ was measured by flow cytometry. The maximum fluorescence intensity was measured by adding calcium ion carrier (1×10^-5^ mmol/L) and CaCl_2_ (1 mmol/L). The fluorescence quenching agent MnCl_2_ (2 mmol/L) was added to measure the minimum fluorescence intensity.

### Measurement of ALP Level Using Gomori Method

The cell suspension of VSMCs was dropped onto the glass slide for 30 min. The slides were placed into the ALP incubation solution and incubated at 37 °C for 12 h and rinsed slightly under water for 2 min. Then the slides were placed into cobalt nitrate solution and incubated at 37 °C for 5 min. After washing with water for 5 min, distilled water was added. The sections were incubated with the vulcanizing solution for 2 min and observed under light microscopy.

### Terminal Deoxynucleotidyl Transferase-Mediated Deoxyuridine Triphosphate Nick-End Labeling Assay

The detection of the apoptosis of VSMC was performed by a TUNEL assay using an *in situ* cell death detection kit (Roche) according to the manufacturer’s instructions. Briefly, the paraffin sections underwent gradient elution with ethanol followed by treatment with Proteinase K solution for 30 min. TUNEL reactive solution was added to the sections and the incubation was fulfilled in a dark wet box at 37 °C for 1 h. After being incubated with converter-POD in the dark at 37 °C for 30 min, the cells were stained with DAB and counterstained with hematoxylin. Imagines were obtained by fluorescence microscope.

### Western Blot

Briefly, cellular proteins of VSMCs were extracted and a BCA kit was applied to measure the concentration of proteins. After that, different groups of protein samples were separated on 10% SDS-PAGE gels (80v, 90min) and then transferred to PVDF membranes (200 mA, 150 min). The membranes were incubated with β-actin (ABclonal, 1:1000), AIF-1 (Abcam, 1:1000), Runx2 (Abcam, 1:1000), α-SMA (ABclonal, 1:1000), NF-κB p65 (CST, 1:1000), CCR-2 (CST, 1:1000), and p-NF-κB p65 (CST, 1:1000) antibodies, respectively, which were diluted at a ratio of 1:1000 with 1X TBST containing 5% BSA. After incubated overnight at 4°C, the membranes were washed three times with TBST and then reacted with a goat anti-rabbit secondary antibody (Beyotime, 1:7500) for 1 h at 37°C. The membranes were washed three times with TBST and once with TBS before being stained with an alkaline phosphatase chromogenic solution. Image J software was performed for analysis of greyscale.

### RT-PCR

Total RNA from aortic ECs was extracted with TRizol reagent (Invitrogen) according to the instructions of the manufacturer. The concentration and purity of RNA were determined with nanodrop by absorbance at 260 nm and 280 nm. And then, complementary DNA was synthesized using the PrimeScript RT reagent kit. Real-time PCR was performed by SYBR Green PCR Master Mix with β-actin as a control. The sequences of the primers for AIF-1, Runx2, α-SMA, and β-actin were shown in **Table 1**.

**Table 1:** Primer sequence for RT-PCR.

Mouse AIF-1-F	5`-GTTTGGACGGCAGATCCTCA-3`
Mouse AIF-1-R	5`-CAGGGATTTGCAGGGAGGAA-3`
Mouse Runx2-F	5′ -AGAGTCAGATTACAGATCCCAGG-3′
Mouse Runx2-R	5′ -AGGAGGGGTAAGACTGGTCATA-3′
Mouse α-SMA-F	5`-AGGGAGTAATGGTTGGAATG-3`
Mouse α-SMA-R	5`-GGTGATGATGGCGTGTTCTAT-3`
Mouse NF-κB p65-F	5`-GAAGCCGCTGACCATGGAA3`
Mouse NF-κB p65-R	5`-GATCACAGCCAAGTGGAGTGGA3`
Mouse CCR-2-F	5`-GTGATTGACAAGCACTTAGAC 3`
Mouse CCR-2-R	5`-ACTCGATCTGCTGTCTCC3`
Mouse β-actin-F	5′-TTCTACAATGAGCTGCGTGT-3′
Mouse β-actin-R	5′-CTCATAGCTCTTCTCCAGGG-3′
Human Runx2-F	5′-AGGGCAGCGAGGTAGTGA-3′
Human Runx2-R	5′-CCTGA AAGCCGATGTGGT-3′
Human α-SMA-F	5′-GGCTATTCCTTCGTGACTACTG-3′
Human α-SMA-R	5′-AGCAG TGGCCATCTCATTT-3′
Human NF-kB p65-F	5′-CCCACGAGCTTGTAGGAAAGG-3′
Human NF-kB p65-R	5′-CTGGATGCGCTGACTGATAG-3′
Human CCR-2-F	5′-CCAACTCCTGCCTCCGCTCTA 3′
Human CCR-2-R	5′-CCGCCAAAATAACCGATGTGATAC 3′
Human β-actin-F	5’-GGCACCCAGCACAATGAA-3’
Human β-actin-R	5’-TAGAAGCATTTGCGGTGG-3’

### Statistical Analysis

Differences between all raw obtained values (mean ± SE) were analyzed by one-way ANOVA for comparisons between multiple groups or by unpaired Student’s t-test for comparisons between two groups using SPSS v21.0 software (IBM, USA). A two-sided P value < 0.05 was considered statistically significant.

## Results

### Overproduction of Aldo and AIF-1 in Vascular Calcification in Murine Renal Failure

**Figure 1:**
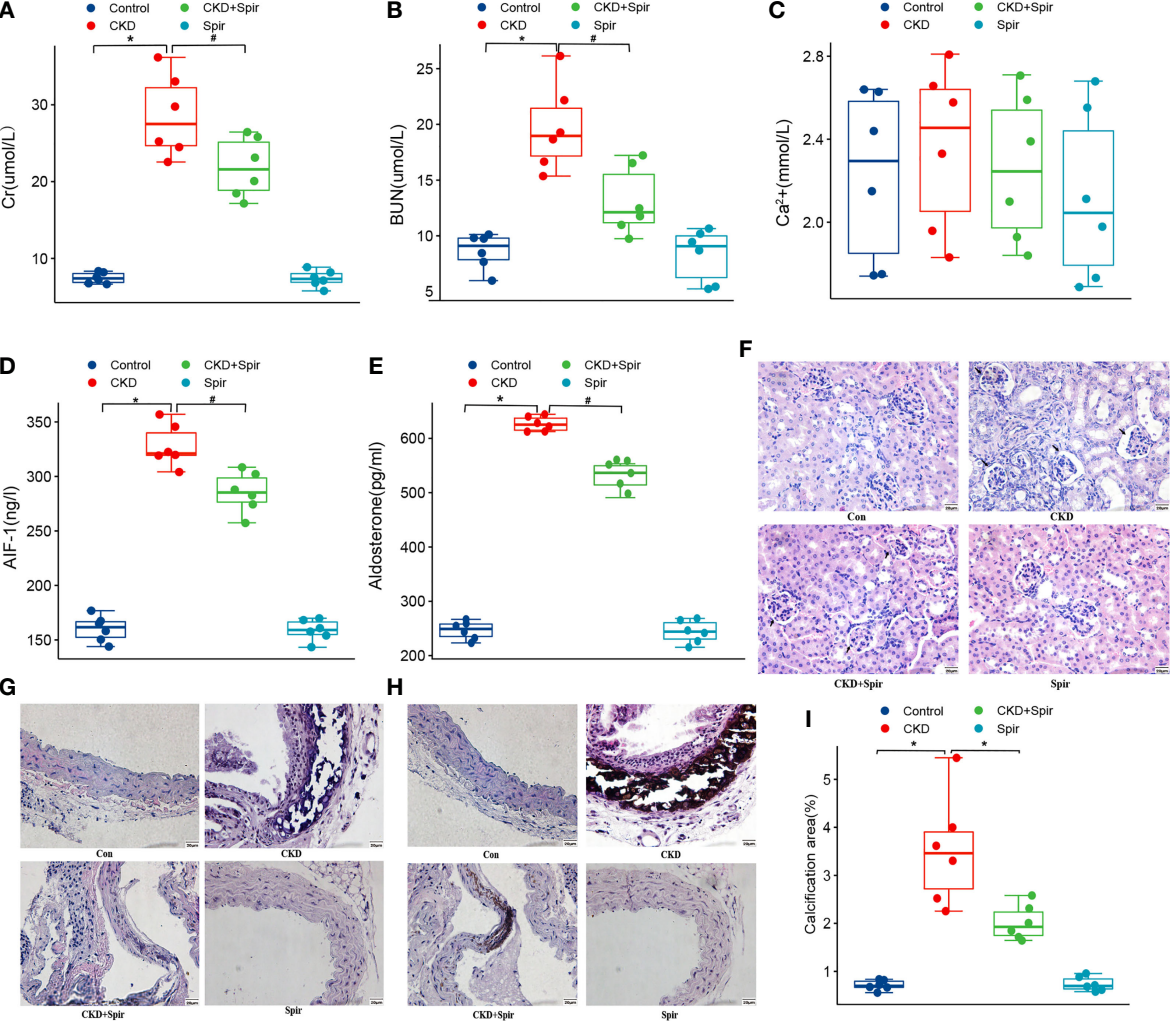
The serum Cr, BUN, Ca^2+^, AIF-1, and Aldo levels in CKD mice model **(A–E)**. The histomorphology of renal tissue(400X) **(F)** and thoracic aorta(400X) **(G)**. VonKossa staining(400X) **(H)** and quantitative analysis **(I)**. (n=6) (*P<0.05, Control group vs. CKD group; ^#^P<0.05, CKD group vs. CKD+Spir group).

Compared with the control group, the Cr, BUN, Aldo, and AIF-1 content in the serum were significantly increased in the CKD group (*P*<0.05), indicating the CKD mice model was established successfully. In addition, compared with the CKD group, the Cr, BUN, Aldo, and AIF-1 content in the serum in the CKD+Spir group have greatly alleviated (P<0.05). Whereas, the Ca^2+^ content in the serum has no significant change among the four groups (**Figures 1A–E**). Besides, under the light microscope, the renal tissue structure of the normal group was normal, and there was no renal tubular dilatation and inflammatory cell infiltration. In the CKD group, the glomerulus tended to atrophy. Brown crystals and protein casts were seen in the cavity, and a large number of black adenine metabolite crystals were deposited. Compared with the CKD group, the CKD+Spir group reduced glomerular, tubular, and interstitial lesions without calcium deposition (**Figure 1F**). Moreover, in the control group, the intima, media, and adventitia of the blood vessels were intact, with a less intercellular matrix. Compared with the CKD group, the lesions in the CKD+Spir group were significantly reduced, with complete vascular structure, mild destruction of the media, and a small amount of patchy deposition (**Figure 1G**). VonKossa staining showed that in the control group, the ECs were intact, the elastic fibers were continuous, and there was no calcium salt deposition; while in the CKD group, the elastic fibers of the thoracic aorta media were ruptured, the intima was damaged, and a large number of dense silver nitrate particles were deposited. And after Spir intervention, the deposition of blue-black particles in the thoracic aorta of mice was reduced, and no obvious calcium nodules were formed (**Figures 1H, I**).

### Calcium and Phosphorus Metabolism in Vascular Calcification in Mouse Renal Failure

**Figure 2:**
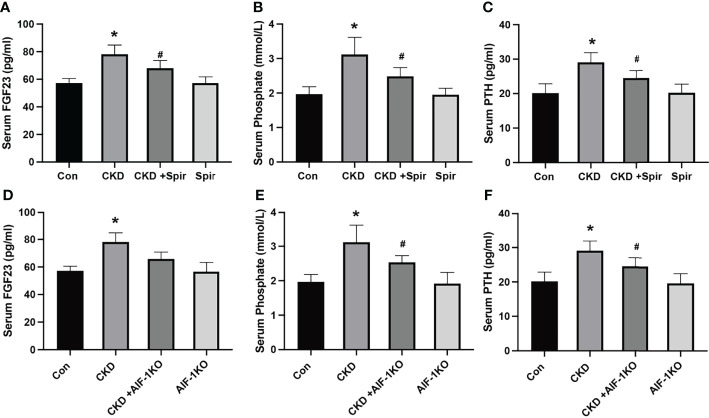
The serum FGF23, Phosphate, and PTH levels in the CKD mice model **(A–C)** and AIF-1KO mice model **(D–F)**. (n=6) (*P<0.05, Control group vs. CKD group; ^#^P<0.05, CKD group vs. CKD+Spir group or CKD group vs. AIF-1KO+CKD group).

The serum levels of FGE23, phosphorus, and PTH were significantly increased in the CKD group compared with the control group (P<0.05). Compared with the CKD group, the serum levels of FGF23, phosphorus, and PTH in the CKD+Spir and CKD+AIF-1KO groups were greatly relieved (P<0.05) (**Figure 2**).

### Increase of Apoptosis, Inflammation, and Osteogenic Transdifferentiation in the Calcified Artery in Murine Renal Failure

**Figure 3:**
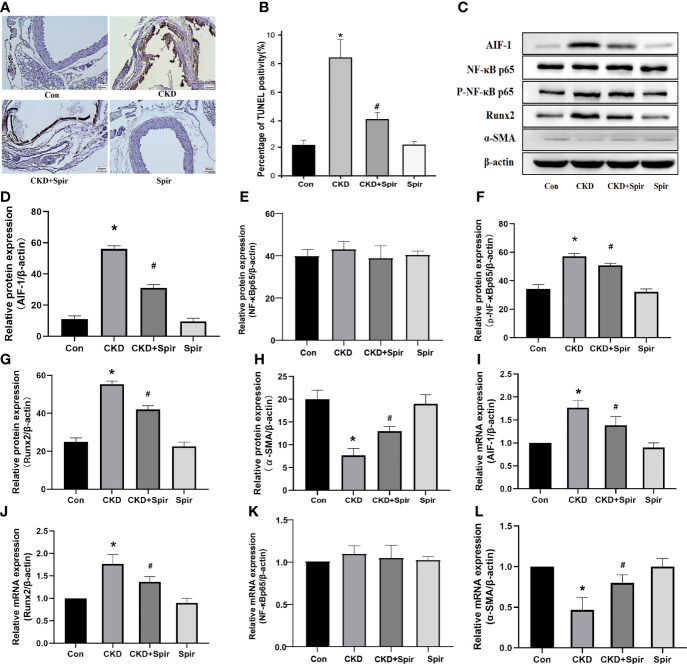
Cell apoptosis in the vascular tissue using TUNEL staining (200X) **(A, B)**. Western blot and PCR analysis **(C–L)**. (n=6) (*P<0.05, Control group vs. CKD group; ^#^P<0.05, CKD group vs. CKD+Spir group).

TUNEL staining results showed that a small amount of cell apoptosis was seen in the vascular tissue of the control group, a large number of brown deposits were seen in the blood vessels of the CKD group mice, and the number of apoptotic cells increased significantly, indicating that Aldo-induced CKD vascular calcification process increased cell apoptosis; after Spir intervention, the number of apoptotic cells was significantly reduced, indicating that Spir can alleviate vascular cell apoptosis in CKD mice (**Figures 3A, B**). Besides, compared with the control group, the expression of Runx2 in the blood vessels of the mice in the CKD group was higher and the expression of α-SMA was lower; compared with the CKD group, the expression of Runx2 was lower, and the expression of α-SMA was higher in the CKD+Spir group, indicating that Spir can reduce the osteogenic transdifferentiation of vascular cells in CKD mice and improve vascular calcification. Compared with the control group, the expressions of AIF-1, CCR2, and p-NF-κB p65 in the blood vessels of the mice in the CKD group were increased, but the change of NF-κB p65 expression was not significant, indicating that Aldo-induced vascular calcification in CKD was accompanied by inflammation and activation of NF-κB p65. After Spir intervention, the expressions of AIF-1, CCR2, and p-NF-κB p65 decreased, indicating that Spir can reduce the inflammatory response of vascular cells in CKD mice (**Figures 3C–L**, **Supplementary Figure 2**).

### Effect of AIF-1 Knockout on Vascular Calcification in Murine Renal Failure

**Figure 4:**
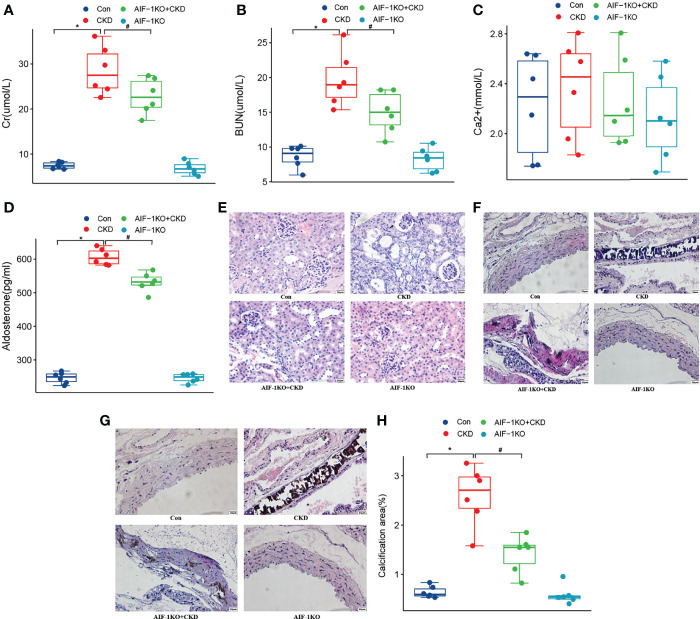
The serum Cr, BUN, Ca^2+^, AIF-1, and Aldo levels in the AIF-1KO mice model **(A–E)**. The histomorphology of renal tissue(400X) **(F)** and thoracic aorta (400X) **(G)**. VonKossa staining(400X) **(H)** and quantitative analysis **(I)**. (n=6) (*P<0.05, Control group vs. CKD group; ^#^P<0.05, CKD group vs. AIF-1KO+CKD group).

Compared with the CKD group, the Cr, BUN, Aldo, and AIF-1 contents in the serum in the AIF-1KO+CKD group have greatly decreased (*P*<0.05), while the serum Ca^2+^ content has no significant change among the four groups (**Figures 4A–D**). Moreover, the histomorphological results showed that compared with the CKD group, glomerular, tubule, and interstitial lesions were reduced and vacuolar degeneration of tubule epithelial cells was observed, and some nuclei were exfoliated without calcium deposits in the AIF-1KO+CKD group (**Figure 4E**). Moreover, compared with the CKD group, the AIF-1KO+CKD group had significantly reduced lesions and less damage to the media (**Figure 4F**). VonKossa staining showed that compared with the CKD group, the AIF-1KO+CKD group had less damage to the middle membrane and has few calcium deposits (**Figures 4G, H**).

### Effect of AIF-1 Knockout on Apoptosis, Inflammation and Osteogenic Transdifferentiation in the Calcified Artery in Murine Renal Failure

**Figure 5:**
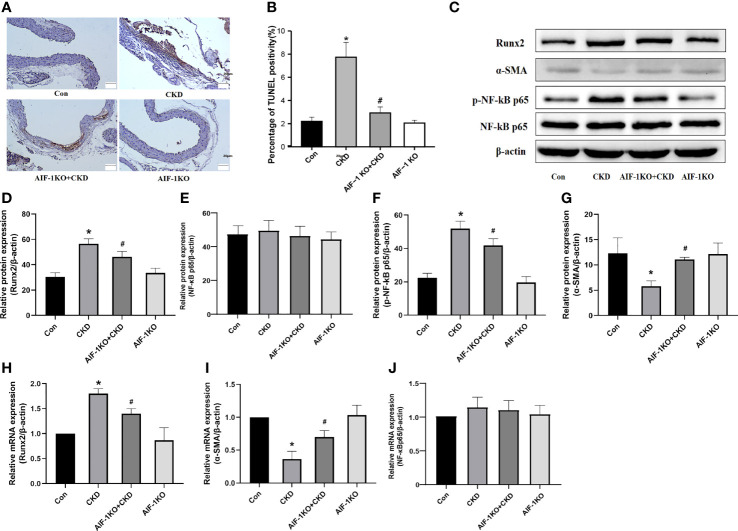
Cell apoptosis in the vascular tissue using TUNEL staining (200X) **(A, B)**. Western blot and PCR analysis **(C–J)**. (n=6) (*P<0.05, Control group vs. CKD group; ^#^P<0.05, CKD group vs. AIF-1KO+CKD group).

The results of TUNEL staining showed that the number of apoptotic cells decreased significantly, indicating that AIF-1 gene knockdown can reduce the apoptosis of vascular cells in CKD mice (**Figures 5A, B**). Besides, compared with the CKD group, Runx2 expression was reduced and α-SMA expression was increased in the AIF-1KO+CKD group. Compared with the CKD group, the expression of p-NF-κB p65 was decreased in the AIF-1KO+CKD group, whereas the expression of NF-κB p65 does not change significantly (**Figures 5C–J**).

### Effects of Silencing AIF-1 in ECs on Aldo-Induced Calcification of VSMCs

**Figure 6:**
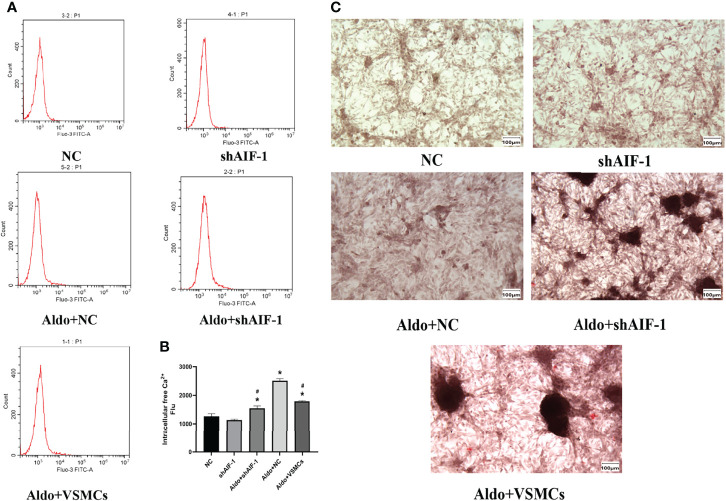
Calcium content using flow cytometric **(A, B)**. The positive area of ALP staining (200X) **(C)**. (n=6) (*P<0.05, NC group vs. Aldo+ NC groups; ^#^P<0.05, Aldo+NC group vs. Aldo+ shAIF-1 groups).

The model of silence AIF-1 in ECs was successfully verified (**Supplementary Figure 1**). Compared with the NC group, the calcium content of VSMCs in the Aldo+NC group was significantly increased (*P*<0.01). Compared with the Aldo+NC group, the calcium content of VSMCs in the Aldo+shAIF-1 group was significantly decreased (*P*<0.01), suggesting that AIF-1 silence in ECs can reduce the Aldo-induced calcium concentration of VSMCs and decrease the Ca^2+^ influx of VSMCs. The calcium content of cells in the Aldo+VSMCs group was lower than that in the Aldo+NC group and higher than that in the Aldo+ shAIF-1 group (**Figures 6A, B**). Besides, compared with the NC group, the positive area of ALP staining of VSMCs in the Aldo+NC group was significantly increased. Compared with the Aldo+NC group, the area of positive ALP staining of VSMCs in the Aldo+shAIF-1 group was significantly lower, indicating that AIF-1 silence in ECs can reduce ALP activity in Aldo-treated VSMCs and decrease osteogenic differentiation. ALP activity in the Aldo+VSMCs group was lower than that in the Aldo+NC group and higher than that in the Aldo+ shAIF-1 group (**Figure 6C**).

### Effects of Silencing AIF-1 in ECs on Aldo-Induced Apoptosis, Inflammation, and Osteogenic Transdifferentiation of VSMCs

**Figure 7:**
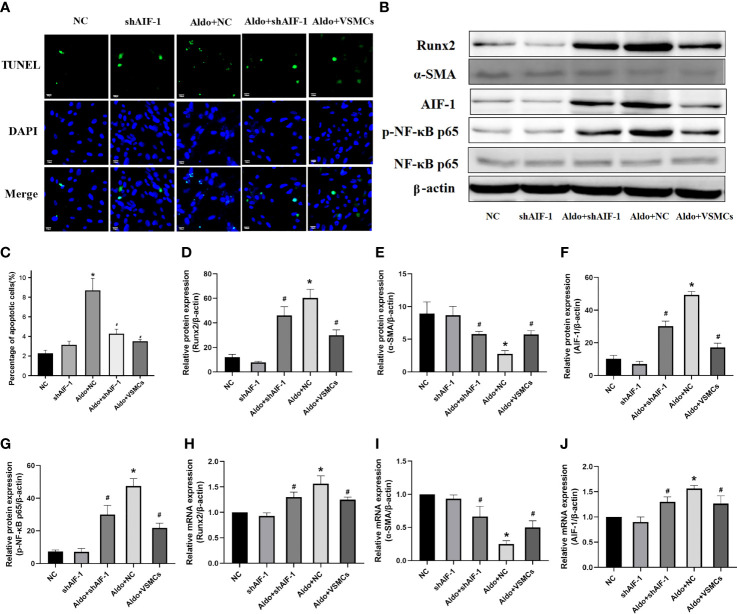
Cell apoptosis using TUNEL analysis (200X) **(A, C)**. Western blot and PCR analysis **(B, D–J)**. (n=6) (*P<0.05, NC group vs. Aldo+NC groups; ^#^P<0.05, Aldo+NC group vs. Aldo+ shAIF-1 groups).

Compared with the NC group, the number of apoptotic cells in VSMCs in the Aldo+NC group was significantly increased. Compared with the Aldo+NC group, the number of apoptotic cells of VSMCs in the Aldo+shAIF-1 group was significantly decreased, suggesting that AIF-1 silence in ECs can reduce the apoptosis of VSMCs induced by Aldo. The number of apoptosis in the Aldo+VSMCs group was lower than that in the Aldo+NC group and higher than that in the Aldo+shAIF-1 group (**Figures 7A, C**). Compared with the NC group, the expression of CCR2 and Runx2 in VSMCs of the Aldo+NC group was increased, and the expression of α-SMA was decreased. Compared with the Aldo+NC group, the expression of Runx2 in VSMCs of the Aldo+shAIF-1 group was decreased, and the expression of α-SMA was increased. The results showed that compared with the NC group, the expression of p-NF-κB p65 in the Aldo+NC group was increased, and the expression of NF-κB p65 had no significant change, indicating that Aldo could induce the activation of NF-κB signaling. Compared with the Aldo+NC group, the expression of p-NF-κB p65 in the Aldo+shAIF-1 group was decreased, indicating that knockdown of AIF-1 could reduce the Aldo-induced inflammatory response and the activation of NF-κB signaling in the co-cultured VSMCs. However, the expression of Runx2 and p-NF-κB p65 in the Aldo+VSMCs group was lower than that of the Aldo+NC group but higher than that of the Aldo+shAIF-1 group, and the expression of α-SMA was higher than that of Aldo+NC group and lower than that of Aldo+shAIF-1 group. (**Figures 7B, D–J**, **Supplementary Figure 2**).

## Discussion

As a pro-inflammatory factor, the AIF-1 signaling pathway may play an important role in the process of cellular calcification. The present study is the first one to demonstrate that AIF-1 was a crucial factor-mediated Aldo-induced osteogenic differentiation and vascular calcification in CKD mice. To explore the mechanism, this study innovatively performed a cell co-culture experiment between ECs and VSMCs to illustrate the network regulation of vascular ECs and VSMCs interaction. And we observed the increased expression of AIF-1 in ECs can directly cause calcium influx and calcification in VSMCs.

Vascular calcification is a major outcome of the transformation of VSMCs into chondroid/osteoblast cells, which was induced by inflammation, disturbance of calcium, apoptosis, and other factors (22). Aldo is a causative agent of vascular calcification, and clinical trials indicated that vascular wall calcification is significantly increased in patients with primary hyperaldosteremia (23). The increased serum Aldo concentration is also associated with cerebral atherosclerosis and cerebrovascular calcification (24). Based on the mice model, we found both the Aldo concentration and AIF-1 expression in the aorta were significantly increased in the CKD group, and Spir (Aldo inhibitor) could reduce the AIF-1 expression. Besides, Spir could also alleviate apoptosis, reduce the expression of α-SMA (myofibroblast marker), CCR2(inflammatory response marker), and increase Runx2 (an early indicator of cellular calcification), thereby improving vascular calcification of CKD. Hence, the results above indicate Aldo can promote inflammation, cell apoptosis, and vascular calcification in the development of CKD.

AIF-1 belongs to the calcium-binding protein containing the Ca^2+^ EF-hand domain (23). As the spatial conformation of AIF-1 is changed, which could activate several pathological processes, such as inflammation, apoptosis, and oxidative stress (25, 26). It was reported that overexpression of AIF-1 in HUVEC could lead to endothelial cell inflammation and oxidative stress through the NF-κB signaling pathway (27). And the increased level of AIF-1 in VSMCs, induced by external stimulation, could also enhance the expression of cyclin and skeleton protein, thus leading to cell migration and phenotypic transformation, atherosclerosis, and vascular inflammation (16). Previous studies showed that inhibition of AIF-1 protein expression in ECs and VSMCs could alleviate atherosclerosis (28–30). And mononuclear macrophage hyperactivation and VSMCs trans-differentiation were observed in AIF-1 over-expressed mice (31). Therefore, AIF-1 is an important inflammatory regulatory factor, which is involved in regulating vascular calcification *via* both ECs and VSMCs.

However, it was still unable to confirm the causal relationship between vascular calcification and AIF-1 expression. In this study, an AIF-1KO mice model was established, and we found that in AIF-1KO+CKD mice, the calcified staining of the aortic wall was significantly attenuated compared to the CKD group, as well as the inflammation response and apoptosis. It was reported that the expressions of MMP2 and MMP-9 in the smooth muscle cells in AIF-1 over-expressed mice were increased after stimulation with oxidized low-density lipoprotein, and the downstream NF-κB signaling pathway was activated (30). Evidence documented that NF-κB could promote the development of vascular calcification in CKD by elevating the environment of phosphorus, calcium, oxidative stress, and inflammation (32). And it also recorded that NF-κB inhibitor could improve the AIF-1-stimulated increase in the secretion of IL-6 and TNF-α inflammatory factors, as well as the oxidative stress state (29). Consistent with other studies, this study firstly found that knockdown of the AIF-1 gene down-regulated the expression of p-NF-κB p65. In addition, AIF-1 could mediate elevations in serum phosphorus, FGF23, PTH, and vascular CCR2 as demonstrated in the present study, which was previously thought to be pathways of NF-κB causing vascular calcification (32). Suggesting that the role of AIF-1 in vascular calcification in CKD may be related to the NF-κB signaling pathway.

Moreover, it is well known that increased local Aldo synthesis could directly induce the increased expression of inflammation-related genes in ECs and VSMCs (33). And the information about endothelial cell injury could be transmitted to smooth muscle cells *via* bypass mode, thus promoting smooth muscle cell synthesis, and inducing inflammation in smooth muscle cells (34). Meanwhile, indirect activation of inflammatory genes could also lead to endothelial cell damage, inducing monocytes to infiltrate into ECs to stimulate the proliferation and differentiation in smooth muscle cells (34). Aldo may be an important factor in initiating and maintaining the interaction among the two types of cells (35). However, there is no evidence of how information is transmitted between them. In our study, we have found the calcification-related indicators were significantly increased in the VSMCs, after stimulating the endothelial cell with Aldo treatment. Under external stimuli, ECs could secrete inflammatory factors, activate the perivascular inflammatory environment (36), and induce osteogenic transdifferentiation and calcification of VSMCs. Another study found that ECs can induce calcification and senescence of VSMCs by releasing exosomes containing calcification-related protein (37). Therefore, we speculated that Aldo may affect the interaction between vascular ECs and VSMCs to communicate calcification. To verify our speculation, we performed co-culture experiments on vascular ECs and VSMCs. The ECs and VSMCs were co-incubated, once the AIF-1 was over-expressed in ECs, the Ca^2+^ influx, and calcification of VSMCs induced by Aldo has exacerbated. On contrary, compared to the CKD group, the Ca^2+^ influx, inflammation response, cell apoptosis, and calcification in the shAIF-1+CKD group were significantly weakened in VSMCs, implying the ECs might transfer AIF-1-mediated inflammatory and calcification signaling to VSMCs through a paracrine form, thereby activating the NF-κB signaling pathway to induce vascular calcification. Therefore, we could conclude that AIF-1 may be an important factor in initiating and maintaining the interaction among the two types of cells.

Our study should be considered alongside a limitation. In our study, in terms of mechanism studies, we detected inflammatory and apoptosis indicators in a targeted way and did not extensively screen the downstream genes of AIF-1. Although we observed that AIF-1 in ECs was a key mediation between Aldo and VSMCs calcification, the effector pathway should be further explored. Therefore, future multiple-omics and mechanism studies should be further performed to systematically explore downstream signaling pathways. Meanwhile, the observations in our study had an important implication. Our study provided a new theoretical basis and a potential therapeutic target for the prevention and treatment of vascular calcification. In terms of clinical medication, we can develop inhibitors of AIF-1 to improve vascular calcification.

In conclusion, Aldo could induce an inflammatory response, apoptosis process, and cellular calcification in VSMCs through the AIF-1 signaling pathway. This would be a potential therapeutic target for vascular calcification induced by chronic kidney disease.

## Data Availability Statement

The original contributions presented in the study are included in the article/**Supplementary Material**. Further inquiries can be directed to the corresponding authors.

## Ethics Statement

The animal study was reviewed and approved by the Animal Ethics Review Committee of Harbin Medical University

## Author Contributions

LH, XW, and XC conceived the idea for the study. XC and JH were involved in the *in vivo* and *in vitro* experiments. XC, JL and JH were involved in the analysis of data. XC and JN wrote the manuscript. All the authors were responsible for revising the manuscript and approved the final version.

## Funding

The present study was supported by the National Natural Science Foundation of China (grant. no. 81870503) and the Heilongjiang Natural Fund joint guiding project (LH2019H025).

## Conflict of Interest

The authors declare that the research was conducted in the absence of any commercial or financial relationships that could be construed as a potential conflict of interest.

## Publisher’s Note

All claims expressed in this article are solely those of the authors and do not necessarily represent those of their affiliated organizations, or those of the publisher, the editors and the reviewers. Any product that may be evaluated in this article, or claim that may be made by its manufacturer, is not guaranteed or endorsed by the publisher.
