# Supplementary material for: The Role of AIF-1 in the Aldosterone-Induced Vascular Calcification Related to Chronic Kidney Disease: Evidence From Mice Model and Cell Co-Culture Model
Source: Front Endocrinol (Lausanne). 2022 Jul 20;13:917356. doi: 10.3389/fendo.2022.917356 (PMC9347268; doi:10.3389/fendo.2022.917356)
Supplement: Supplementary file 1 [file DataSheet_1.pdf]

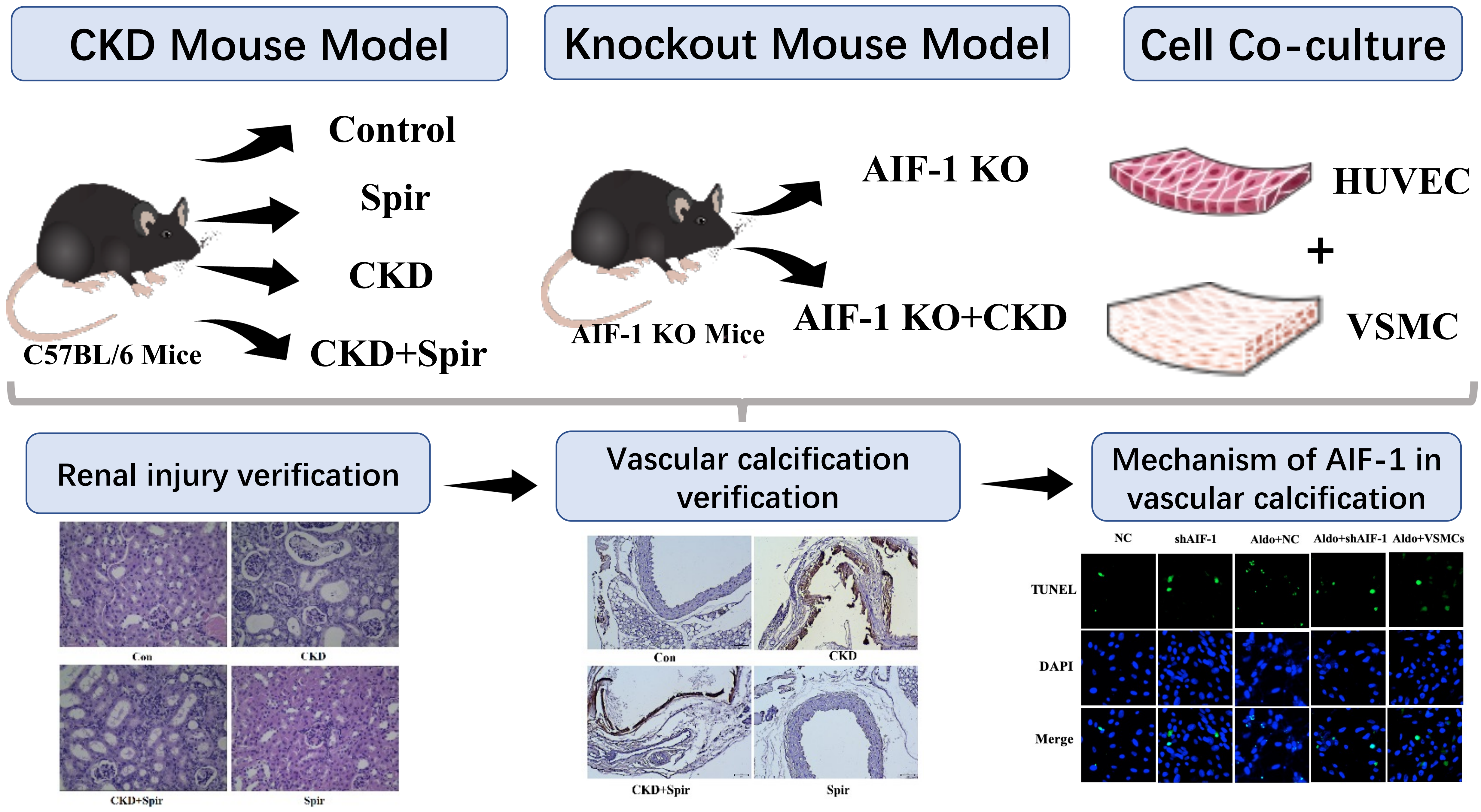
CKD Mouse Model
Knockout Mouse Model
Cell Co-culture
Control
Spir
CKD
CKD+Spir
C57BL/6 Mice
AIF-1 KO
AIF-1 KO+CKD
AIF-1 KO Mice
HUVEC
+
VSMC
Renal injury verification
Vascular calcification verification
Mechanism of AIF-1 in vascular calcification
Con
CKD
CKD+Spir
Spir
Con
CKD
CKD+Spir
Spir
NC
shAIF-1
Aldo+NC
Aldo+shAIF-1
Aldo+VSMCs
TUNEL
DAPI
Merge
