# Supplementary figures and images for: The Role of AIF-1 in the Aldosterone-Induced Vascular Calcification Related to Chronic Kidney Disease: Evidence From Mice Model and Cell Co-Culture Model
Source: Front Endocrinol (Lausanne). 2022 Jul 20;13:917356. doi: 10.3389/fendo.2022.917356 (PMC9347268; doi:10.3389/fendo.2022.917356)

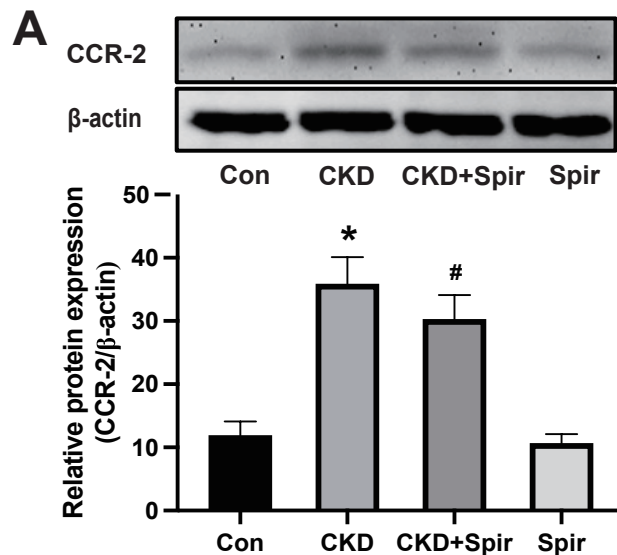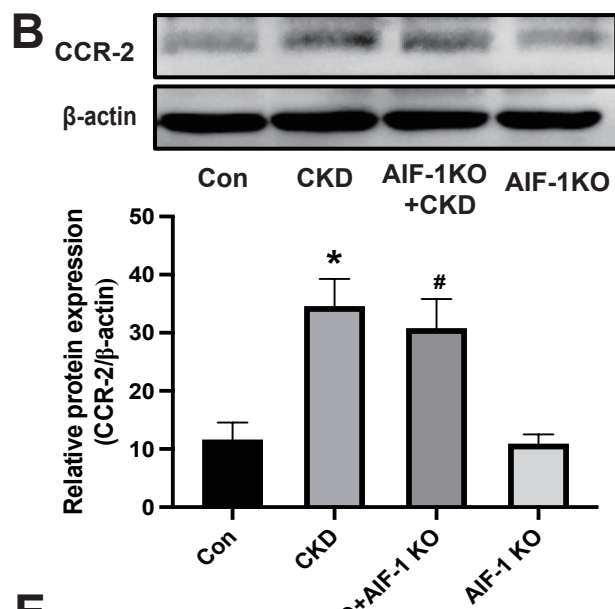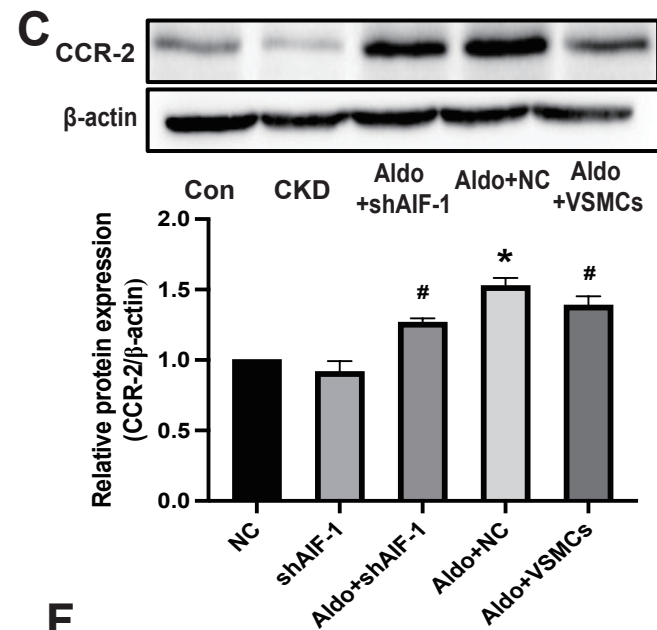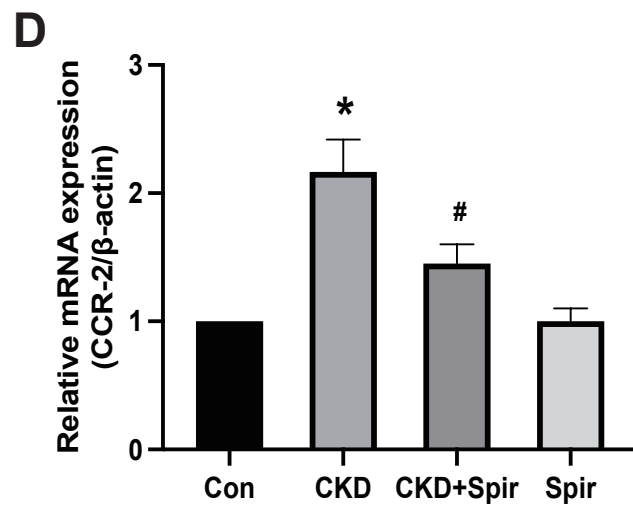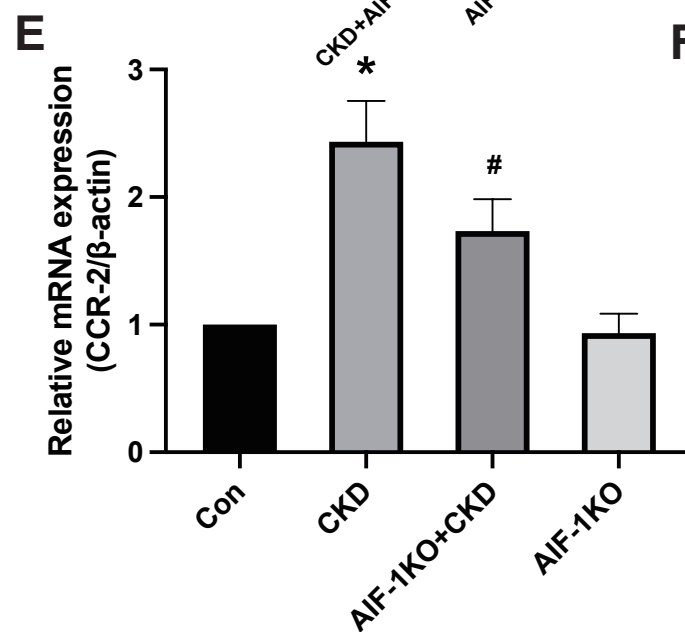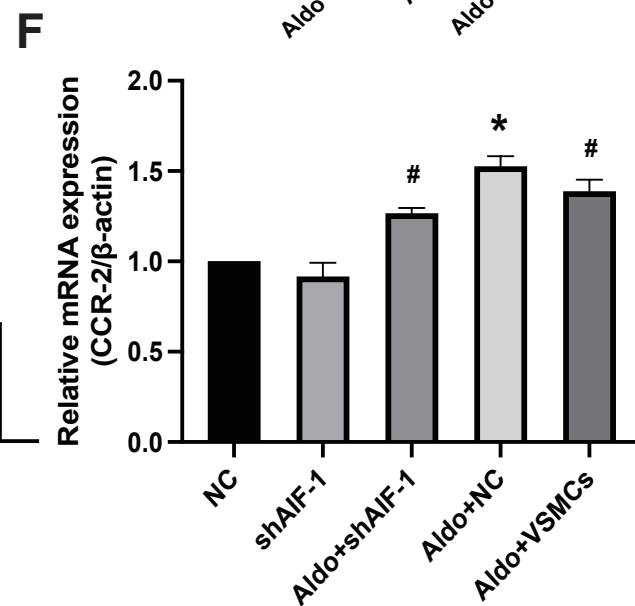

Supplement: Supplementary file 2 [file DataSheet_2.pdf]

AIF-1  
silence

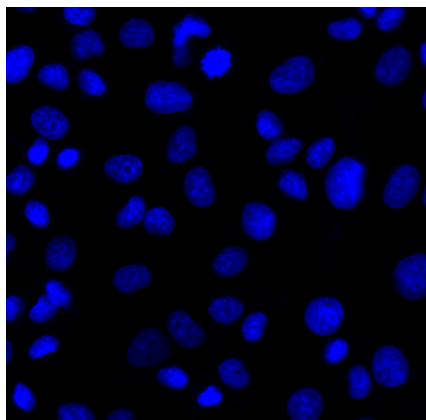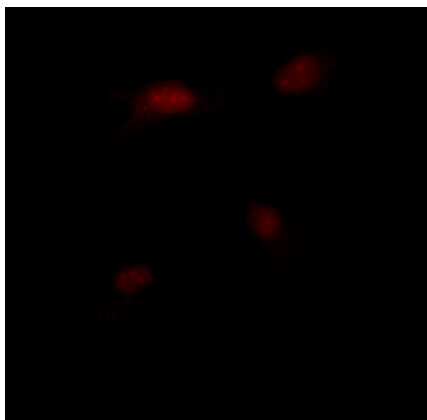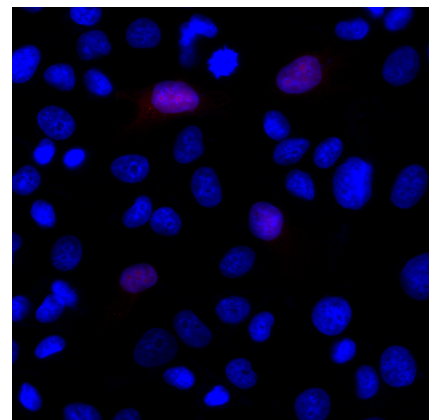

Control  
group

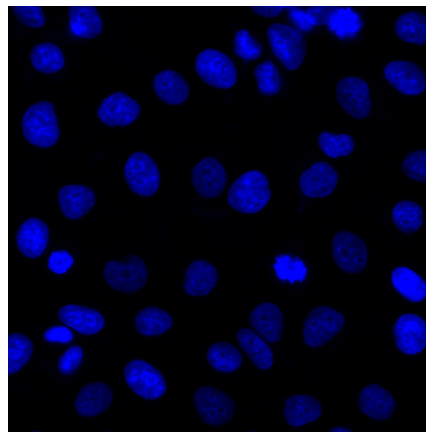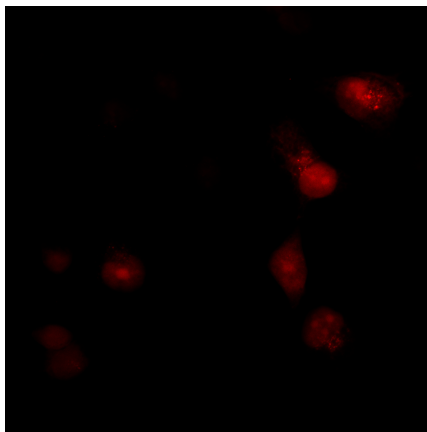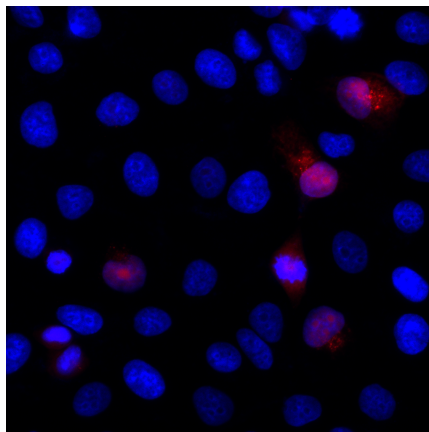

DAP1

AIF-1

Merge

Supplement: Supplementary file 3 [file DataSheet_3.pdf]
