# Supplementary material for: The Role of AIF-1 in the Aldosterone-Induced Vascular Calcification Related to Chronic Kidney Disease: Evidence From Mice Model and Cell Co-Culture Model
Source: Front Endocrinol (Lausanne). 2022 Jul 20;13:917356. doi: 10.3389/fendo.2022.917356 (PMC9347268; doi:10.3389/fendo.2022.917356)
Supplement: Supplementary file 4 [file Table_1.docx]

| Reagent and Resource Source Identifier | | |
| --- | --- | --- |
| Antibodies | | |
| Rabbit monoclonal Anti-AIF-1 | Abcam | Cat#ab32516; |
| Rabbit monoclonal Anti-RUNX2 | Abcam | Cat#ab236639; |
| Mouse monoclonal Anti-NF-κB p65 | Cell Signaling Technology | Cat#6956 |
| Mouse monoclonal Anti-p-NF-κB p65 | Cell Signaling Technology | Cat#3039 |
| Mouse polyclonal Anti- α-SMA | ABclonal | A7248 |
| Rabbit monoclonal Anti-β-actin | ABclonal | AC026 |
| Chemicals | | |
| Fetal bovine serum | [Gibco](https://www.thermofisher.cn/cn/zh/home/brands/gibco.html),Thermo Fisher Scientific | NA |
| Dulbecco's modified Eagle's medium | [Gibco](https://www.thermofisher.cn/cn/zh/home/brands/gibco.html),Thermo Fisher Scientific | NA |
| Aldosterone | Sigma Aldrich | NA |
| Spironolactone | Sigma Aldrich | NA |
| Real-time PCR related reagents | Tak ara Bio Inc | NA |
| Critical commercial assays | | |
| AIF-1 ELISA kit | Shanghai Langdon Biotechn- ology Co., Ltd | NA |
| Alkaline phosphat ase staining kit | Beijing Solarbio Technology Co., Ltd. | G1481 |
| Fluo-3 AM kit | Shanghai Biyuntian Biotech- nology Co., Ltd. | NA |
| Experimental models: organisms | | |
| C57BL/6J | Beijing Vital River Laboratory Animal Technology Co., Ltd | NA |
| AIF- 1KO | Center for Biochemistry and Molecular Biology in Harbin Medical University | NA |
| Experimental models: cell lines |  |  |
| VSMCs | Beina Chuanglian Biotechnology Co., Ltd | KCB200644YJ |
| ECs | American Type Culture Collection |  |
| Other |  |  |
| Adenine feed | Beijing Keao Xieli Feed Co., Ltd | NA |
| Standard feed | Beijing Keao Xieli Feed Co., Ltd | NA |
